# Safety and Outcomes of Combined Pancreatic and Hepatic Resections for Metastatic Pancreatic Neuroendocrine Tumors

**DOI:** 10.1245/s10434-022-12029-7

**Published:** 2022-06-22

**Authors:** Hallbera Gudmundsdottir, Ron Pery, Rondell P. Graham, Cornelius A. Thiels, Susanne G. Warner, Rory L. Smoot, Mark J. Truty, Michael L. Kendrick, Thorvardur R. Halfdanarson, Elizabeth B. Habermann, David M. Nagorney, Sean P. Cleary

**Affiliations:** 1 Department of Surgery, Mayo Clinic, Rochester, Minnesota USA; 2 Robert D. and Patricia E. Kern Center for the Science of Health Care Delivery, Mayo Clinic, Rochester, Minnesota USA; 3 Department of Laboratory Medicine and Pathology, Mayo Clinic, Rochester, Minnesota USA; 4 Department of Medical Oncology, Mayo Clinic, Rochester, Minnesota USA

## Abstract

**Background:**

Approximately 40–50% of patients with pancreatic neuroendocrine tumors (pNETs) initially present with distant metastases. Little is known about the outcomes of patients undergoing combined pancreatic and hepatic resections for this indication.

**Methods:**

Patients who underwent hepatectomy for metastatic pNETs at Mayo Clinic Rochester from 2000 to 2020 were retrospectively reviewed. Major pancreatectomy was defined as pancreaticoduodenectomy or total pancreatectomy, and major hepatectomy as right hepatectomy or trisegmentectomy. Characteristics and outcomes of patients who underwent pancreatectomy with simultaneous hepatectomy were compared with those of patients who underwent isolated hepatectomy (with or without prior history of pancreatectomy).

**Results:**

205 patients who underwent hepatectomy for metastatic pNETs were identified: 131 underwent pancreatectomy with simultaneous hepatectomy and 74 underwent isolated hepatectomy. Among patients undergoing simultaneous hepatectomy, 89 patients underwent minor pancreatectomy with minor hepatectomy, 11 patients underwent major pancreatectomy with minor hepatectomy, 30 patients underwent minor pancreatectomy with major hepatectomy, and 1 patient underwent major pancreatectomy with major hepatectomy. Patients undergoing simultaneous hepatectomy had more numerous liver lesions (10 or more lesions in 54% vs. 34%, *p* = 0.008), but the groups were otherwise similar. Rates of any major complications (31% versus 24%, *p* = 0.43), hepatectomy-specific complications such as bile leak, hemorrhage, and liver failure (0.8–7.6% vs. 1.4–12%, *p* = 0.30–0.99), and 90-day mortality (1.5% vs. 2.7%, *p* = 0.62) were similar between the two groups. 5-year overall survival was 64% after combined resections and 65% after isolated hepatectomy (*p* = 0.93).

**Conclusion:**

For patients with metastatic pNETs, combined pancreatic and hepatic resections can be performed with acceptable morbidity and mortality in selected patients at high-volume institutions.

Pancreatic neuroendocrine tumors (pNETs) are rare tumors, representing only about 1–2% of pancreatic malignancies, but their incidence has significantly increased in the last few decades, particularly for smaller early stage tumors.^1,2^ Although pNETs usually exhibit more indolent behavior than pancreatic adenocarcinoma, distant metastases are evident in 40–50% of patients at the time of initial diagnosis.^2,3^ The presence of distant metastases is one of the strongest predictors of poor prognosis, with an overall 5-year survival of approximately 20–40% in this group.^4–6^ Interestingly, even in the presence of distant metastases, primary tumor resection has been associated with a possible survival benefit.^7,8^ Distant metastases are most commonly found in the liver, involved in about 90% of cases, and debulking hepatectomy has not only been associated with a survival benefit, but may also provide symptomatic relief in patients with hormonally functional tumors.^9–12^ Historically, debulking hepatectomy was recommended only when at least 90% of hepatic disease could be resected, but more recent studies have suggested that lowering this threshold to 70% may also be of benefit.^13,14^

Current consensus guidelines from the North American Neuroendocrine Tumor Society (NANETS) state that data on the safety of combined pancreatectomy and hepatectomy in patients presenting with pNETs and synchronous liver metastases are lacking.^15^ While several studies have reported the short-term outcomes of simultaneous resection of any primary neuroendocrine tumor and liver metastases, the majority of primary tumors included in these studies were of small intestinal origin, the resection of which carries a vastly different risk profile compared with pancreatectomy.^14,16,17^ Similarly, while several studies have reported the short-term outcomes of patients undergoing simultaneous pancreatectomy and hepatectomy for various indications, only a minority were performed for pNETs.^18–24^

Compared with other indications for combined pancreatectomy and hepatectomy, patients with metastatic pNETs undergo different types of both pancreatic and hepatic resections, have higher complication rates after pancreatectomy, and have an overall better prognosis.^20,25^ Therefore, dedicated studies in this particular population are needed. The aim of this study was to provide data on the safety of pancreatectomy with simultaneous hepatectomy in patients with metastatic pNETs by evaluating our institutional experience with these resections and comparing short-term outcomes with those of patients undergoing isolated hepatectomy for pNET liver metastases.

## Methods

The study was approved by the Mayo Clinic Institutional Review Board. Patients who underwent hepatectomy for metastatic pNETs at Mayo Clinic Rochester from January 2000 to December 2020 were identified. Clinical data were obtained from medical records. Simultaneous hepatectomy was defined as hepatectomy performed with curative or debulking intent at the same time as primary tumor resection. Isolated hepatectomy was defined as hepatectomy without concurrent pancreatectomy (with or without prior history of pancreatectomy). Extent of resection was estimated based on operative notes, including descriptions of intraoperative ultrasound, and by comparing pre- and postoperative cross-sectional imaging. Extent of resection was categorized as 70–90% or > 90%, and patients who underwent resection of less than 70% of hepatic disease were excluded. Patients who underwent resection of all visible disease were considered to have undergone > 90% debulking, recognizing that even in this group, resection is almost never complete due to microscopic lesions that are unable to be visualized on preoperative or intraoperative imaging.^26^ Major pancreatectomy was defined as pancreaticoduodenectomy or total pancreatectomy, and minor pancreatectomy as distal pancreatectomy or enucleation. Major hepatectomy was defined as right hepatectomy or trisegmentectomy (with or without minor resections of the contralateral lobe) and minor hepatectomy as all other types of resections based on data from the National Surgical Quality Improvement Program (NSQIP).^27^

Severity of comorbidities was calculated according to the Charlson Comorbidity Index (CCI).^28^ As all patients in this study had metastatic cancer, the lowest possible CCI score was 6. Patients were considered to have a hereditary cancer syndrome if they had positive genetic testing or met the best available clinical criteria for one of the following syndromes associated with pNET formation: multiple endocrine neoplasia type 1, von Hippel-Lindau, tuberous sclerosis, or neurofibromatosis type 1.^29–32^ Tumor grade was assigned according to the 2019 World Health Organization (WHO) classification of neuroendocrine neoplasms of the digestive system in cases where Ki-67 and/or mitotic count were available. If neither Ki-67 nor mitotic count were available, the grade assigned by the reviewing pathologist was used. The Clavien-Dindo system was used to classify postoperative complications occurring within 90 days of surgery and major complications were defined as Clavien-Dindo ≥ 3.^33^ Pancreatectomy-specific complications [postoperative pancreatic fistula (POPF), post-pancreatectomy hemorrhage (PPH), delayed gastric emptying (DGE)] and hepatectomy-specific complications [post-hepatectomy bile leakage (PHBL), post-hepatectomy hemorrhage (PHH), post-hepatectomy liver failure (PHLF)] were defined and graded according to the respective International Study Group of Pancreatic Surgery (ISGPS) and International Study Group of Liver Surgery (ISGLS) classifications.^34–39^ For patients undergoing simultaneous pancreatectomy and hepatectomy, PPH and PHH were combined into one complication due to overlap in diagnostic criteria.

For statistical analysis, chi-square or Fisher’s exact tests were utilized for comparing categorical variables and Mann-Whitney *U* test or Kruskal-Wallis one-way analysis of variance for comparing the medians of continuous variables. Overall and progression-free survival were estimated according to the Kaplan-Meier method and differences observed among patient subgroups were assessed by the log-rank test. Overall survival was calculated from the date of surgery to the date of death or to the date of most recent follow-up for non-deceased patients. Progression was defined as findings on imaging consistent with recurrence or increased tumor burden with or without pathologic confirmation. Progression-free survival was calculated from the date of surgery to the date of progression or to the date of most recent follow-up for patients who did not have progression. Two-sided *p*-values were computed and *p* < 0.05 was considered statistically significant. All statistical calculations were performed using R (version 4.0.0).

## Results

### Short-Term Outcomes After Simultaneous Versus Isolated Hepatectomy

**Fig. 1.**
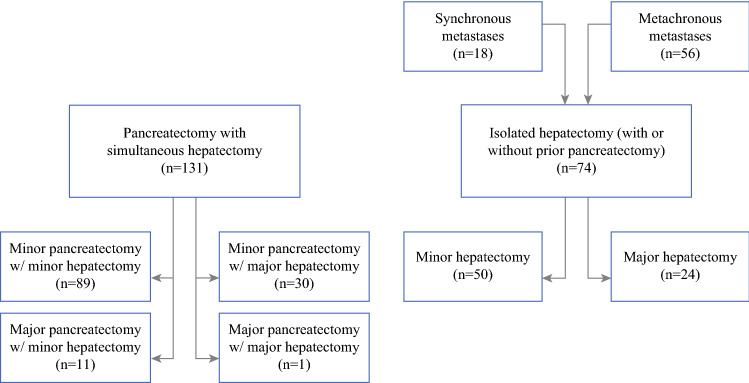
Study cohort. Diagram showing the number of patients in the simultaneous versus isolated hepatectomy groups, as well as the different combinations of pancreatectomy and/or hepatectomy for each group

From 2000 to 2020, 131 patients underwent pancreatectomy with simultaneous hepatectomy and 74 patients (18 with synchronous metastases and 56 with metachronous metastases) underwent isolated hepatectomy for metastatic pNETs at Mayo Clinic Rochester (Fig. 1). Of the 74 patients who underwent isolated hepatectomy, 67 had previously undergone resection of the primary tumor. The remaining seven patients underwent hepatectomy for symptomatic disease (*n* = 2) or debulking purposes only (*n* = 5), while the pancreatic tumors were left in place due to unresectability or necessitating an extensive resection not considered worth pursuing in the presence of metastatic disease. The characteristics of patients who underwent simultaneous versus isolated hepatectomy are shown in Table 1. No significant difference was found in age, sex, race, comorbidity index, tumor functionality, presence of a hereditary cancer syndrome, Ki-67 index, overall tumor grade, or length of follow-up. Patients in the simultaneous hepatectomy group were more likely to have 10 or more liver lesions (54% versus 34%, *p* = 0.008), but no significant difference was found in largest lesion size (median 42 versus 34 mm, *p* = 0.26) or prevalence of extrahepatic disease (8.4% versus 8.1%, *p* = 0.99).

**Table 1.** Characteristics of patients who underwent pancreatectomy with simultaneous hepatectomy compared with those who underwent isolated hepatectomy for synchronous or metachronous metastases

	Simultaneous hepatectomy (*n* = 131)	Isolated hepatectomy (*n* = 74)	*p*-value
Age (years)	56 (45–64)	57 (50–63)	0.62
Female sex	59 (45%)	36 (49%)	0.72
White race^a^	120 (93%)	70 (96%)	0.54
Charlson Comorbidity Index	7 (6–8)	8 (7–8)	0.14
Functional tumor	26 (20%)	12 (16%)	0.65
Hereditary cancer syndrome	10 (7.6%)	7 (13%)	0.39
Ki-67 ≥3%^a^	58 (84%)	24 (71%)	0.18
Overall grade ≥2^a^	86 (76%)	45 (67%)	0.26
10+ liver metastases	71 (54%)	25 (34%)	0.008
Largest liver lesion (mm)	42 (22–65)	34 (19–61)	0.26
Extrahepatic disease	11 (8.4%)	6 (8.1%)	0.99
Follow-up (months)	48 (24–97)	57 (19–118)	0.81

Categorical variables are shown as number (percentage) and continuous variables as median (interquartile range)

^a^Patients with missing information on race (*n* = 3), Ki-67 (*n* = 102), and grade (*n* = 25) were excluded from the respective analyses.

Major hepatectomy was performed in 55 patients (27%): 35 of 108 patients (32%) in the earlier half of the study period and 20 of 97 patients (21%) in the latter half (*p* = 0.08). Overall, major hepatectomy was performed in 31 patients (24%) in the simultaneous hepatectomy group and 24 patients (32%) in the isolated hepatectomy group (*p* = 0.23). Resection of > 90% of hepatic disease was achieved in 123 patients (94%) in the simultaneous hepatectomy group and 72 patients (97%) in the isolated hepatectomy group (*p* = 0.34). A minimally invasive approach was utilized in 8 patients (6.1%) in the simultaneous hepatectomy group and 5 patients (6.8%) in the isolated hepatectomy group (*p* = 0.99). Intraoperative ablation was used in combination with resection in 58 patients (44%) in the simultaneous hepatectomy group and 26 patients (35%) in the isolated hepatectomy group (*p* = 0.26). Perioperative outcomes of patients who underwent simultaneous versus isolated hepatectomy are shown in Table 2. Patients undergoing simultaneous hepatectomy had longer operative times (median 290 versus 219 minutes, *p* < 0.001), higher estimated blood loss (> 1000 ml in 34% versus 12%, *p* = 0.002), and a higher rate of transfusions intraoperatively or in the first 72 h after surgery (44% versus 27%, *p* = 0.028). The simultaneous hepatectomy group had longer lengths of stay than the isolated hepatectomy group (median 7 versus 6 days, *p* < 0.001), but when compared with the combined length of stay following both pancreatectomy and hepatectomy in the isolated hepatectomy group, median length of stay was shorter after simultaneous hepatectomy (median 7 versus 14 days, *p* < 0.001). No significant difference was found in the rates of any major complications (31% versus 24%, *p* = 0.43), grade B-C hepatectomy-specific complications such as PHBL (6.9% versus 12%, *p* = 0.30), PPH/PHH (7.6% versus 5.4%, *p* = 0.77), and PHLF (0.8% versus 1.4%, *p* = 0.99), or unplanned reoperations (5.3% versus 8.1%, *p* = 0.63). Overall, mortality was low and similar across groups at 30 days (0.8% versus 2.7%, *p* = 0.30) and 90 days (1.5% versus 2.7%, *p* = 0.62) from surgery.

**Table 2.** Perioperative outcomes of patients who underwent pancreatectomy with simultaneous hepatectomy compared with those who underwent isolated hepatectomy

	Simultaneous hepatectomy (*n* = 131)	Isolated hepatectomy (*n* = 74)	*p*-value
Major hepatectomy	31 (24%)	24 (32%)	0.23
Operative time (minutes)^a^	290 (247–366)	219 (171–262)	< 0.001
Estimated blood loss >1000 ml^a^	44 (34%)	8 (12%)	0.002
Transfusion within 72 hours	57 (44%)	20 (27%)	0.028
Major complications (CD ≥3)	40 (31%)	18 (24%)	0.43
PHBL (grades B-C)	9 (6.9%)	9 (12%)	0.30
PPH or PHH (grades B-C)	10 (7.6%)	4 (5.4%)	0.77
PHLF (grades B-C)	1 (0.8%)	1 (1.4%)	0.99
Unplanned reoperation	7 (5.3%)	6 (8.1%)	0.63
Length of stay (days)	7 (6-11)	6 (4–8)	< 0.001
30-day mortality	1 (0.8%)	2 (2.7%)	0.30
90-day mortality	2 (1.5%)	2 (2.7%)	0.62

Categorical variables are shown as number (percentage) and continuous variables as median (interquartile range)

*CD*, Clavien-Dindo; *PHBL*, post-hepatectomy bile leakage; *PPH*, post-pancreatectomy hemorrhage; *PHH*, post-hepatectomy hemorrhage; *PHLF*, post-hepatectomy liver failure

^a^Patients with missing information on operative time (*n* = 2) and estimated blood loss (*n* = 8) were excluded from the respective analyses

### Short-Term Outcomes After Different Combinations of Simultaneous Pancreatectomy and Hepatectomy

Of the 131 patients who underwent pancreatectomy with simultaneous hepatectomy, 89 patients (70%) underwent minor pancreatectomy with minor hepatectomy, 11 patients (8.4%) major pancreatectomy with minor hepatectomy, 30 patients (23%) minor pancreatectomy with major hepatectomy, and 1 patient (0.8%) major pancreatectomy with major hepatectomy (Fig. 1). Patient characteristics of the first three groups are shown in Table 3. No significant difference was found in age, sex, race, comorbidity index, tumor functionality, presence of a hereditary cancer syndrome, primary tumor size, Ki-67 index, overall tumor grade, or length of follow-up. The median size of the largest liver lesion was 35 mm in patients who underwent minor pancreatectomy with minor hepatectomy, 22 mm in those who underwent major pancreatectomy with minor hepatectomy, and 65 mm in those who underwent minor pancreatectomy with major hepatectomy (*p* < 0.001). No significant difference was found in number of liver lesions (10 or more lesions in 27–63%, *p* = 0.12) or prevalence of extrahepatic disease (0.0–17%, *p* = 0.19) between the three groups.

**Table 3.** Characteristics of patients who underwent pancreatectomy with simultaneous hepatectomy stratified by types of procedures. One patient who underwent major pancreatectomy with major hepatectomy was excluded from comparison

	Minor pancreatectomy with minor hepatectomy (*n* = 89)	Major pancreatectomy with minor hepatectomy (*n* = 11)	Minor pancreatectomy with major hepatectomy (*n* = 30)	*p*-value
Age (years)	58 (46–63)	59 (51–66)	53 (44–66)	0.71
Female sex	41 (46%)	4 (36%)	13 (43%)	0.85
White race^a^	82 (93%)	11 (100%)	26 (90%)	0.63
Charlson Comorbidity Index	7 (6–8)	8 (7–8)	7 (6–8)	0.56
Functional tumor	16 (18%)	3 (27%)	7 (23%)	0.56
Hereditary cancer syndrome	6 (6.7%)	2 (18%)	2 (6.7%)	0.34
Primary tumor size (mm)	46 (29–70)	40 (38–49)	47 (31–74)	0.79
Ki-67 ≥3%^a^	38 (86%)	5 (71%)	14 (82%)	0.54
Overall grade ≥2^a^	60 (77%)	6 (75%)	19 (73%)	0.93
10+ liver metastases	48 (54%)	3 (27%)	19 (63%)	0.12
Largest liver lesion (mm)	35 (20–60)	22 (14–28)	65 (56–107)	<0.001
Extrahepatic disease	6 (6.7%)	0 (0.0%)	5 (17%)	0.19
Follow up (months)	48 (21–97)	38 (19–72)	53 (38–87)	0.33

Categorical variables are shown as number (percentage) and continuous variables as median (interquartile range)

^a^Patients with missing information on race (*n* = 2), Ki-67 (*n* = 62), and grade (*n* = 18) were excluded from the respective analyses

Perioperative outcomes of patients who underwent pancreatectomy with simultaneous hepatectomy stratified by type of procedure are shown in Table 4. No significant difference was observed for median operative time, frequency of estimated blood loss > 1000 ml, or rate of transfusion intraoperatively or within 72 h from surgery between the three groups. The rate of grade B–C PPH or PHH was 4.5% after minor pancreatectomy with minor hepatectomy, 27% after major pancreatectomy with minor hepatectomy, and 10% after minor pancreatectomy with major hepatectomy (*p* = 0.031). No significant difference was observed for the rate of any major complications (26–46%, *p* = 0.18) or other grade B-C hepatectomy- or pancreatectomy-specific complications such as PHBL (0.0–17%, *p* = 0.07), PHLF (0.0–3.3%, *p* = 0.32), POPF (17–27%, *p* = 0.48), and DGE (4.5–18%, *p* = 0.07). Additionally, rates of unplanned reoperation were similar (4.5–9.1%, *p* = 0.53). Median length of stay was 7 days after minor pancreatectomy with minor hepatectomy, 13 days after major pancreatectomy with minor hepatectomy, and 12 days after minor pancreatectomy with major hepatectomy (*p* = 0.001). No significant difference was observed in the rate of mortality at 30 days (0.0–1.1%, *p* = 0.99) or 90 days (0.0–9.1%, *p* = 0.21) from surgery.

**Table 4.** Perioperative outcomes of patients who underwent pancreatectomy with simultaneous hepatectomy stratified by types of procedures

	Minor pancreatectomy with minor hepatectomy (*n*=89)	Major pancreatectomy with minor hepatectomy (*n* = 11)	Minor pancreatectomy with major hepatectomy (*n* = 30)	*p*-value
Operative time (minutes)	286 (248–361)	349 (294–457)	273 (224–360)	0.07
Estimated blood loss >1000 ml	26 (29%)	6 (55%)	12 (40%)	0.18
Transfusion within 72 hours	40 (45%)	4 (36%)	12 (40%)	0.81
Major complications (CD ≥3)	23 (26%)	5 (46%)	12 (40%)	0.18
PHBL (grades B-C)	4 (4.5%)	0 (0.0%)	5 (17%)	0.07
PPH or PHH (grades B-C)	4 (4.5%)	3 (27%)	3 (10%)	0.031
PHLF (grades B-C)	0 (0.0%)	0 (0.0%)	1 (3.3%)	0.32
POPF (grades B-C)	15 (17%)	2 (20%)^a^	8 (27%)	0.48
DGE (grades B-C)	4 (4.5%)	2 (18%)	4 (13%)	0.07
Unplanned reoperation	4 (4.5%)	1 (9.1%)	2 (6.7%)	0.53
Length of stay (days)	7 (6–8)	13 (6–17)	12 (7–17)	0.001
30-day mortality	1 (1.1%)	0 (0.0%)	0 (0.0%)	0.99
90-day mortality	1 (1.1%)	1 (9.1%)	0 (0.0%)	0.21

One patient who underwent major pancreatectomy with major hepatectomy was excluded from the comparison

Categorical variables are shown as number (percentage) and continuous variables as median (interquartile range).

*CD*, Clavien-Dindo; *PHBL*, post-hepatectomy bile leakage; *PPH*, post-pancreatectomy hemorrhage; *PHH*, post-hepatectomy hemorrhage; *PHLF*, post-hepatectomy liver failure; *POPF*, postoperative pancreatic fistula; *DGE*, delayed gastric emptying

^a^Patients who underwent total pancreatectomy (*n* = 1) were excluded from the POPF denominator

### Long-Term Outcomes After Simultaneous Versus Isolated Hepatectomy

**Fig. 2.**
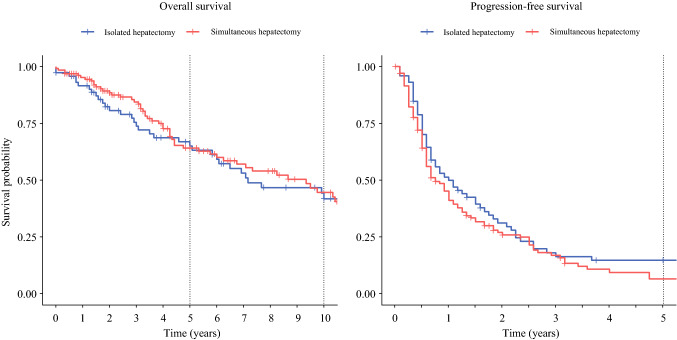
Kaplan-Meier curves for overall and progression-free survival after simultaneous pancreatectomy and hepatectomy (*n* = 131) and isolated hepatectomy (*n* = 74). Survival curves were similar for overall (*p* = 0.93) and progression-free (*p* = 0.21) survival

Five-year overall survival was 64% (95% CI 55–75) after simultaneous hepatectomy and 65% (95% CI 54–78) after isolated hepatectomy, and 10-year overall survival was 45% (95% CI 34–58) after simultaneous hepatectomy and 42% (95% CI 30–58) after isolated hepatectomy. Median overall survival was 9.3 years (95% CI 6.3–12.3) after simultaneous hepatectomy and 7.2 years (95% CI 6.0–13.2) after isolated hepatectomy. Two-year progression-free survival was 26% (95% CI 19–35) after simultaneous hepatectomy and 31% (95% CI 22–45) after isolated hepatectomy, and 5-year progression-free survival was 7% (95% CI 3–15) after simultaneous hepatectomy and 15% (95% CI 8–27) after isolated hepatectomy. Median progression-free survival was 9.0 months (95% CI 7.0–13.0) after simultaneous hepatectomy and 12 months (95% CI 8.0–20.0) after isolated hepatectomy. Kaplan-Meier survival curves are shown in Fig. 2 and were similar for overall (*p* = 0.93) and progression-free (*p* = 0.21) survival. In total, 29 patients (14%) went on to have a second hepatectomy: 14 (11%) in the simultaneous hepatectomy group and 15 (20%) in the isolated hepatectomy group (*p* = 0.09).

## Discussion

pNETs with synchronous liver metastases are frequently encountered and optimal management in patients with resectable disease involves both resection of the primary pancreatic tumor and debulking hepatectomy to improve survival, as well as to provide symptomatic relief in the case of functional disease.^10–15^ In most circumstances, the preferred approach at our institution has been to address both the pancreas and liver during the same operation, therefore sparing the patient a second operation. However, little has been published about the safety of these combined procedures. In this study, we report a 21-year institutional experience of performing pancreatectomy with simultaneous hepatectomy for metastatic pNETs and compare outcomes with those of patients who underwent isolated hepatectomy (with or without prior history of pancreatectomy) for the same indication.

Several studies have reported the outcomes of patients undergoing combined pancreatic and hepatic resections for either biliary tract malignancies only or for any indication, including pNETs and a variety of locally aggressive non-hepatobiliary intra-abdominal malignancies.^18–23^ Those that included patients undergoing distal pancreatectomy with any type of hepatectomy or pancreaticoduodenectomy with minor hepatectomy generally reported acceptable morbidity and mortality rates following these procedures, but unacceptably high rates after combined pancreaticoduodenectomy and major hepatectomy, with perioperative mortality rates of up to 21%. The one study to focus specifically on combined pancreatic and hepatic resections in patients with metastatic pNETs was published by our group in 2002 and described the outcomes of 23 patients who underwent distal pancreatectomy with either minor or major hepatectomy in the pre-2000 era.^40^ In that cohort, major complications occurred in 18% of patients and there was no perioperative mortality.

In the present study, we further expand on our institutional data, including not only distal pancreatectomies but all types of pancreatic resections, and add a comparison with patients undergoing isolated hepatectomy for the same indication. We found that compared with isolated hepatectomy, pancreatectomy with simultaneous hepatectomy was associated with longer operative times, higher estimated blood loss, and higher transfusion rates. Patients who underwent isolated hepatectomy had slightly higher, although not statistically significant, rates of hepatectomy-specific complications, unplanned reoperations, and perioperative mortality, which is likely due to the slightly higher rate of major hepatectomy in this group. Patients who underwent simultaneous hepatectomy had slightly longer lengths of stay following surgery, but when compared with the combined length of stay in patients who underwent both isolated pancreatectomy and isolated hepatectomy, overall length of stay was significantly shorter when the procedures were combined. Both groups had good long-term survival with 5-year overall survival rates of approximately 65% from surgery, which is similar to what has previously been reported, and important when considering aggressive resections for patients with metastatic disease.^12,13^ While both groups demonstrated good overall survival following surgery, the 5-year progression-free survival rate was only 7–15%, demonstrating that although these resections can improve symptoms and prolong survival, they are almost never curative. Although survival rates were similar, care should be taken when comparing these groups as all patients in the simultaneous hepatectomy group had synchronous metastases while patients in the isolated hepatectomy group had a mixture of synchronous and metachronous metastatic disease

The majority of our combined pancreatectomy and hepatectomy cohort underwent minor pancreatectomy (distal pancreatectomy or enucleation) with either minor or major hepatectomy. A small subset underwent major pancreatectomy (pancreaticoduodenectomy or total pancreatectomy) with minor hepatectomy, but only one patient underwent combined major pancreatectomy and major hepatectomy. At our institution, patients requiring both major pancreatectomy and major hepatectomy are typically managed with staged procedures, which is supported by several studies reporting poor outcomes when these procedures have been combined for other indications.^18–20^ When the different procedure combinations were compared, we observed a significantly higher rate of PPH or PHH after major pancreatectomy with minor hepatectomy compared with other combinations. Similarly, the highest rates of any major complication and 90-day mortality were seen after major pancreatectomy with minor hepatectomy, although these differences were not statistically significant, possibly due to the small size of some of the subgroups. Similar trends in overall complication and perioperative mortality rates were observed by Tran et al. in a recent analysis of combined pancreatic and hepatic resections for all indications in the NSQIP database.^19^ This suggests that for patients undergoing combined pancreatic and hepatic resections, short-term outcomes are more significantly affected by the extent of pancreatic resection rather than the extent of hepatic resection. This can be contrasted with data on combined primary tumor and hepatic resections for metastatic colorectal cancer, where both institutional and NSQIP data have shown that short-term outcomes are more significantly affected by the extent of hepatic resection.^26,41^

The study has several limitations. The retrospective single-center design, with all resections performed at a center with extensive experience with these operations, means that our conclusions may not be applicable to every institution. Furthermore, as Ki-67 was rarely reported prior to the publication of the 2010 WHO classification for neuroendocrine neoplasms of the digestive system, Ki-67 data is missing in approximately half of the study cohort and overall grade reported in those patients may not be fully comparable with the grading system currently in use. Lastly, as all patients in the simultaneous hepatectomy group had synchronous metastatic disease but the isolated hepatectomy group included patients with both synchronous and metachronous metastases, we acknowledge that these groups may not be fully comparable. While we believe that short-term outcomes can appropriately be compared between the two groups, this must be considered when comparing long-term survival.

## Conclusion

In conclusion, combining minor hepatectomy with any type of pancreatectomy and major hepatectomy with minor pancreatectomy is safe and should be considered in all patients presenting with pNETs with synchronous liver metastases and resectable disease. Although not supported directly by our data, we believe that patients who require both major pancreatectomy and major hepatectomy are best managed by staged procedures.

## References

[1] Halfdanarson TR, Rabe KG, Rubin J, Petersen GM (2008). Pancreatic neuroendocrine tumors (PNETs): incidence, prognosis and recent trend toward improved survival. Ann Oncol..

[2] Sonbol MB, Mazza GL, Oliver T (2022). Survival and incidence patterns of pancreatic neuroendocrine tumors over the last two decades: a SEER database analysis. Oncologist..

[3] Boyar Cetinkaya R, Aagnes B, Thiis-Evensen E, Tretli S, Bergestuen DS, Hansen S (2017). Trends in incidence of neuroendocrine neoplasms in Norway: a report of 16,075 cases from 1993 through 2010. Neuroendocrinology..

[4] Bilimoria KY, Talamonti MS, Tomlinson JS (2008). Prognostic score predicting survival after resection of pancreatic neuroendocrine tumors: analysis of 3851 patients. Ann Surg..

[5] Birnbaum DJ, Turrini O, Ewald J (2014). Pancreatic neuroendocrine tumor: a multivariate analysis of factors influencing survival. Eur J Surg Oncol..

[6] Ekeblad S, Skogseid B, Dunder K, Oberg K, Eriksson B (2008). Prognostic factors and survival in 324 patients with pancreatic endocrine tumor treated at a single institution. Clin Cancer Res..

[7] Tao L, Xiu D, Sadula A (2017). Surgical resection of primary tumor improves survival of pancreatic neuroendocrine tumor with liver metastases. Oncotarget..

[8] Shaib WL, Zakka K, Penley M (2021). Role of resection of the primary in metastatic well-differentiated neuroendocrine tumors. Pancreas..

[9] Riihimäki M, Hemminki A, Sundquist K, Sundquist J, Hemminki K (2016). The epidemiology of metastases in neuroendocrine tumors. Int J Cancer..

[10] Osborne DA, Zervos EE, Strosberg J (2006). Improved outcome with cytoreduction versus embolization for symptomatic hepatic metastases of carcinoid and neuroendocrine tumors. Ann Surg Oncol..

[11] Mayo SC, de Jong MC, Bloomston M (2011). Surgery versus intra-arterial therapy for neuroendocrine liver metastasis: a multicenter international analysis. Ann Surg Oncol..

[12] Fairweather M, Swanson R, Wang J (2017). Management of neuroendocrine tumor liver metastases: long-term outcomes and prognostic factors from a large prospective database. Ann Surg Oncol..

[13] Woltering EA, Voros BA, Beyer DT (2017). Aggressive surgical approach to the management of neuroendocrine tumors: a report of 1,000 surgical cytoreductions by a single institution. J Am Coll Surg..

[14] Maxwell JE, Sherman SK, O’Dorisio TM, Bellizzi AM, Howe JR (2016). Liver-directed surgery of neuroendocrine metastases: What is the optimal strategy?. Surgery..

[15] Howe JR, Merchant NB, Conrad C (2020). The North American Neuroendocrine Tumor Society consensus paper on the surgical management of pancreatic neuroendocrine tumors. Pancreas..

[16] Glazer ES, Tseng JF, Al-Refaie W (2010). Long-term survival after surgical management of neuroendocrine hepatic metastases. HPB (Oxford)..

[17] Gaujoux S, Gonen M, Tang L (2012). Synchronous resection of primary and liver metastases for neuroendocrine tumors. Ann Surg Oncol..

[18] D'Angelica M, Martin RC, Jarnagin WR, Fong Y, DeMatteo RP, Blumgart LH (2004). Major hepatectomy with simultaneous pancreatectomy for advanced hepatobiliary cancer. J Am Coll Surg..

[19] Tran TB, Dua MM, Spain DA, Visser BC, Norton JA, Poultsides GA (2015). Hepato-pancreatectomy: how morbid? Results from the national surgical quality improvement project. HPB (Oxford)..

[20] Hemming AW, Magliocca JF, Fujita S (2010). Combined resection of the liver and pancreas for malignancy. J Am Coll Surg..

[21] Nimura Y, Hayakawa N, Kamiya J (1991). Hepatopancreatoduodenectomy for advanced carcinoma of the biliary tract. Hepatogastroenterology..

[22] Wakai T, Shirai Y, Tsuchiya Y, Nomura T, Akazawa K, Hatakeyama K (2008). Combined major hepatectomy and pancreaticoduodenectomy for locally advanced biliary carcinoma: long-term results. World J Surg..

[23] Ebata T, Yokoyama Y, Igami T (2012). Hepatopancreatoduodenectomy for cholangiocarcinoma: a single-center review of 85 consecutive patients. Ann Surg..

[24] Addeo P, Oussoultzogiou E, Fuchshuber P (2014). Safety and outcome of combined liver and pancreatic resections. Br J Surg..

[25] Partelli S, Tamburrino D, Cherif R (2019). Risk and predictors of postoperative morbidity and mortality after pancreaticoduodenectomy for pancreatic neuroendocrine neoplasms: a comparative study with pancreatic ductal adenocarcinoma. Pancreas..

[26] Elias D, Lefevre JH, Duvillard P (2010). Hepatic metastases from neuroendocrine tumors with a “thin slice” pathological examination: they are many more than you think. Ann Surg..

[27] Shubert CR, Habermann EB, Bergquist JR (2015). A NSQIP review of major morbidity and mortality of synchronous liver resection for colorectal metastasis stratified by extent of liver resection and type of colorectal resection. J Gastrointest Surg..

[28] Charlson ME, Pompei P, Ales KL, MacKenzie CR (1987). A new method of classifying prognostic comorbidity in longitudinal studies: development and validation. J Chronic Dis..

[29] Thakker RV, Newey PJ, Walls GV (2012). Clinical practice guidelines for multiple endocrine neoplasia type 1 (MEN1). J Clin Endocrinol Metab..

[30] Maher ER, Neumann HP, Richard S (2011). von Hippel-Lindau disease: a clinical and scientific review. Eur J Hum Genet..

[31] Northrup H, Krueger DA (2013). Tuberous sclerosis complex diagnostic criteria update: recommendations of the 2012 International Tuberous Sclerosis Complex Consensus Conference. Pediatr Neurol..

[32] National Institutes of Health (1988). National Institutes of Health Consensus Development Conference statement: neurofibromatosis. Neurofibromatosis..

[33] Dindo D, Demartines N, Clavien PA (2004). Classification of surgical complications: a new proposal with evaluation in a cohort of 6336 patients and results of a survey. Ann Surg..

[34] Bassi C, Marchegiani G, Dervenis C (2017). The 2016 update of the International Study Group (ISGPS) definition and grading of postoperative pancreatic fistula: 11 years after. Surgery..

[35] Wente MN, Veit JA, Bassi C (2007). Postpancreatectomy hemorrhage (PPH): an International Study Group of Pancreatic Surgery (ISGPS) definition. Surgery..

[36] Wente MN, Bassi C, Dervenis C (2007). Delayed gastric emptying (DGE) after pancreatic surgery: a suggested definition by the International Study Group of Pancreatic Surgery (ISGPS). Surgery..

[37] Koch M, Garden OJ, Padbury R (2011). Bile leakage after hepatobiliary and pancreatic surgery: a definition and grading of severity by the International Study Group of Liver Surgery. Surgery..

[38] Rahbari NN, Garden OJ, Padbury R (2011). Post-hepatectomy haemorrhage: a definition and grading by the International Study Group of Liver Surgery (ISGLS). HPB (Oxford)..

[39] Rahbari NN, Garden OJ, Padbury R (2011). Posthepatectomy liver failure: a definition and grading by the International Study Group of Liver Surgery (ISGLS). Surgery..

[40] Sarmiento JM, Que FG, Grant CS, Thompson GB, Farnell MB, Nagorney DM (2002). Concurrent resections of pancreatic islet cell cancers with synchronous hepatic metastases: outcomes of an aggressive approach. Surgery..

[41] Driedger MR, Yamashita TS, Starlinger P (2021). Synchronous resection of colorectal cancer primary and liver metastases: an outcomes analysis. HPB (Oxford)..

